# Supplementary material for: Digital Ecology: Coexistence and Domination among Interacting Networks
Source: Sci Rep. 2015 May 19;5:10268. doi: 10.1038/srep10268 (PMC4437301; doi:10.1038/srep10268)
Supplement: Supplementary Information [file srep10268-s1.pdf]

Supplementary Information for:
Digital Ecology: Coexistence and Domination among Interacting Networks

Kaj-Kolja Kleineberg[1] and Marián Boguñá[1]

[1] *Departament de Física Fonamental, Universitat de Barcelona, Martí i Franquès 1, 08028 Barcelona, Spain*

(Dated: April 7, 2015)

Keywords: digital ecology — complex systems — complex networks — interacting networks — social networks — coexistence — preferential attachment — diminishing returns

Numerical simulations

To simulate our model we use the Pokec network [1], a real OSN, as the underlying off-line network. We take advantage of the fact that the temporal events in our model are independent Poisson point processes, which allows us to use the Gillespie [2, 3] algorithm (also known as the Doob-Gillespie [4] algorithm). For a single network, the algorithm works as follows:

1. Initialize the system and fix the rates corresponding to the respective events (here λ, μ, $\delta = 1$)

2. (a) Evaluate the number of susceptible nodes (N_S), the number of active nodes (N_A) as well as the number of edges connecting susceptible and active nodes (E_{SA}) and the number of edges connecting active and passive nodes (E_{PA}). Evaluate the sum $\mathcal{S} = \mu N_S + \lambda [E_{SA} + E_{PA}] + \delta N_A$.

   (b) Generate random numbers to choose the next event. The probabilities for the events are the following:
   - Mass media activation: $\mu N_S / \mathcal{S}$
   - Viral activation: $\lambda E_{SA} / \mathcal{S}$
   - Viral reactivation: $\lambda E_{PA} / \mathcal{S}$
   - Deactivation: $\delta N_A / \mathcal{S}$

   (c) Evaluate the corresponding time step τ. The corresponding time step is given by $\tau = \mathcal{S}^{-1}$.

3. Update the status of the system. So, if in step 2 a mass media activation was chosen, we randomly choose a susceptible node and change its status to active. For a deactivation, we randomly choose an active node which then becomes passive. In the case of viral activation, we randomly choose an edge connecting a susceptible and an active node and activate the susceptible node at the end of the link. Similarly, for viral reactivation we randomly choose an edge connecting a passive and an active node and the passive node at the end of this edge becomes active. We increase the time: $t \to t + \tau$. We iterate by returning to step 2 until the end of the simulation is reached.

Generalization of the algorithm to multiple layers is straightforward. One evaluates the probabilities of having a certain dynamical process in a certain layer; for example, mass media activation in layer i occurs with probability $\mu_i N_{S,i}$, where $N_{S,i}$ denotes the number of susceptible nodes with respect to layer i (all the nodes which are in the underlying network but not in the i-th layer) and μ_i is the corresponding rate in layer i. Accordingly, the probability of viral activation in layer i is given by $\lambda_i E_{SA,i}$, where $E_{SA,i}$ is the number of edges connecting active and susceptible nodes in layer i. One then chooses a dynamical processes in a certain layer in accordance with these probabilities. Finally, τ is given by the inverse of the sum over all these probabilities in all the layers.

Independent of the topological properties of the network, the activities for the steady state solution for an arbitrary number of layers is encoded in the activity curve of a single layer, which we show in Fig. 1. At the steady state of n_c coexisting networks, each prevailing layer has the same share of the total virality $\lambda_i = \omega_i \lambda = \frac{\lambda}{n_c}$, whereas the remaining ones have $\lambda_j = 0$. The steady state activity of the i-th network is then given by the activity value of a single layer shown in Fig. 1 at $\lambda = \lambda_i$.

Jacobian matrix

Here we analyze the Jacobian matrix of the dynamical system defined in the paper whose entries correspond to

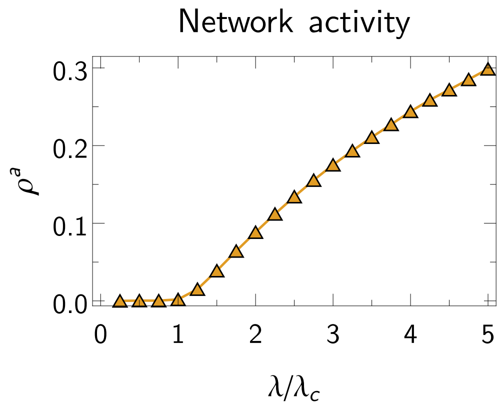

Figure 1: Steady state activity of our model for a single network when using Pokec as the underlying off-line network as a function of the virality parameter λ (see [1]). Below a critical value λ_c, the network becomes entirely passive.

the following derivatives

$$\begin{aligned}
\left.\frac{\partial\dot{\rho}_i^a}{\partial\rho_j^a}\right|_{\boldsymbol{\rho}^*} &= \rho_i^{a*} n_l \left.\frac{\partial\omega_i(\boldsymbol{\rho}^a)}{\partial\rho_j^a}\right|_{\boldsymbol{\rho}^*} - \delta_{ij}\frac{\rho_i^{a*}}{1-\rho_i^{a*}} \\
\left.\frac{\partial\dot{\rho}_i^a}{\partial\rho_j^s}\right|_{\boldsymbol{\rho}^*} &= \delta_{ij}\frac{\lambda}{\nu n_l} \\
\left.\frac{\partial\dot{\rho}_i^s}{\partial\rho_j^a}\right|_{\boldsymbol{\rho}^*} &= 0 \\
\left.\frac{\partial\dot{\rho}_i^s}{\partial\rho_j^s}\right|_{\boldsymbol{\rho}^*} &= -\delta_{ij}\left[\frac{\lambda}{\nu n_l} + \frac{\rho_i^{a*}}{1-\rho_i^{a*}}\right].
\end{aligned} \tag{1}$$

The Jacobian matrix (of dimension $2n_l \times 2n_l$) can be written as

$$\mathcal{J} = \begin{pmatrix} \mathcal{M}_{a,a} & \mathcal{M}_{a,s} \\ \mathcal{M}_{s,a} & \mathcal{M}_{s,s} \end{pmatrix} \tag{2}$$

where $\mathcal{M}_{\eta,\theta}$ represent $n_l \times n_l$ matrices with the following elements

$$\mathcal{M}_{\eta,\theta}(i,j) = \left.\frac{\partial\dot{\rho}_i^\eta}{\partial\rho_j^\theta}\right|_{\boldsymbol{\rho}^*}. \tag{3}$$

The matrix $\mathcal{M}_{s,s}$ is a diagonal matrix with all its diagonal equal to $d = -\left[\frac{\lambda}{\nu n_l} + \frac{\rho_i^{a*}}{1-\rho_i^{a*}}\right] < 0$. $\mathcal{M}_{a,s}$ is also a diagonal matrix with all its diagonal elements equal to some value c and finally $\mathcal{M}_{s,a}$ has all its elements equal to 0.

By using the Laplace expansion starting from the $2n_l, 2n_l$ entry and expanding row-wise, one finds after n_l iterations that

$$\det\left[\mathcal{J} - \Lambda\mathcal{I}\right] = (d-\Lambda)^{n_l}\det\left[\mathcal{M}_{a,a} - \Lambda\mathcal{I}\right] \tag{4}$$

which means we have the eigenvalues $\Lambda_{n_l+1,\dots,2n_l} = d$ with degeneracy n_l and the remaining eigenvalues are those of $\mathcal{M}_{a,a}$. Because $d<0$, this means that the stability of the coexistence solution is exclusively determined by the dynamics in the limit $\nu \to \infty$, which reduces the dimensionality of the system from $2n_l$ to n_l and decouples the dynamics of ρ_i^a from ρ_i^s. See Fig. 4 in the paper for the dynamical properties.

The matrix $\mathcal{M}_{a,a}$ has the form

$$\mathcal{M}_{a,a} = \begin{pmatrix} \alpha & \beta & \cdots & \beta \\ \beta & \alpha & \cdots & \beta \\ \vdots & \vdots & \ddots & \vdots \\ \beta & \beta & \cdots & \alpha \end{pmatrix} \tag{5}$$

and its eigenvalues are $\Lambda_1 = \alpha + (n_l - 1)\beta$ and $\Lambda_{2,\dots,n_l} = (\alpha - \beta)$, which has degeneracy $n_l - 1$. With

$$\alpha = \rho_i^{a*} n_l \left.\frac{\partial\omega_i(\boldsymbol{\rho}^a)}{\partial\rho_i^a}\right|_{\boldsymbol{\rho}^*} - \frac{\rho_i^{a*}}{1-\rho_i^{a*}} \tag{6}$$

and

$$\beta = \rho_i^{a*} n_l \left.\frac{\partial\omega_i(\boldsymbol{\rho}^a)}{\partial\rho_j^a}\right|_{\boldsymbol{\rho}^*} = -\rho_i^{a*}\frac{n_l}{n_l-1}\left.\frac{\partial\omega_i(\boldsymbol{\rho}^a)}{\partial\rho_i^a}\right|_{\boldsymbol{\rho}^*} \tag{7}$$

(here we used $\sum_j \omega_j = 1$, hence $\frac{\partial\omega_i}{\partial\rho_j^a}|_{i\neq j} = -\frac{1}{n_l-1}\frac{\partial\omega_i}{\partial\rho_i^a}$). From here, one obtains the result that Λ_1 is always negative and the stability is controlled by the eigenvalues $\Lambda_{2,\dots,n_l}$, which are

$$\Lambda_{2,\dots,n_l} = \alpha - \beta = \left.\frac{\partial\omega_i(\boldsymbol{\rho}^a)}{\partial\rho_i^a}\right|_{\boldsymbol{\rho}^*} \rho_i^{a*}\frac{n_l^2}{n_l-1} - \frac{\rho_i^{a*}}{1-\rho_i^{a*}} \tag{8}$$

which have to be negative to satisfy stable coexistence. This leads to the condition

$$\frac{n_l^2}{n_l-1}(1-\rho_i^{a*})\left.\frac{\partial\omega_i(\boldsymbol{\rho}^a)}{\partial\rho_i^a}\right|_{\boldsymbol{\rho}^{a*}} < 1 \tag{9}$$

as described in Eq. (6) in the paper.

Dynamical response to a small perturbation for $n \gg 1$

To evaluate the stability of the system in the coexistence state, one has to perform an analysis of the Jacobian matrix as shown in section . In this section, we discuss the response of the system to a small perturbation, $\Delta\rho_i^a$, of the activity of one network. In the limit $n \gg 1$, we can neglect the effect of perturbing the i-th network on the remaining ones. The perturbation induces a shift in the corresponding weight according to

$$\Delta\omega_i \approx \left.\frac{\partial\omega_i}{\partial\rho_i^a}\right|_{\boldsymbol{\rho}^{a*}} \Delta\rho_i^a. \tag{10}$$

Our initial perturbation triggers the dynamical response $\Delta\tilde{\rho}$ from the system given by

$$\Delta\tilde{\rho}_i^a = \left.\frac{\partial\rho_i^{a*}(\omega_i)}{\partial\omega_i}\right|_{\omega_i=\frac{1}{n_l}} \Delta\omega_i, \tag{11}$$

where $\rho_i^{a*}(\omega_i) = 1 - 1/\lambda\langle k\rangle\omega_i$. With Eq. (10), we obtain

$$\Delta\tilde{\rho}_i^a = n_l(1-\rho_i^{a*})\left.\frac{\partial\omega_i}{\partial\rho_i^a}\right|_{\boldsymbol{\rho}^{a*}}\Delta\rho_i^a. \tag{12}$$

The coexistence solution is stable if the perturbation decreases; this means that the dynamical response $\Delta\tilde{\rho}_i^a$ has to be smaller than the initial perturbation $\Delta\rho_i^a$. Mathematically, this leads to the condition

$$n_l(1-\rho_i^{a*})\left.\frac{\partial\omega_i}{\partial\rho_i^a}\right|_{\boldsymbol{\rho}^{a*}} < 1 \tag{13}$$

which is equivalent to Eq. (6) in the paper in the limit $n_l \gg 1$. The left-hand side of Eq. (13) is proportional the ratio between the dynamical response of the system and the initial perturbation. If this ratio is smaller than one, the initial perturbation will decrease and the coexistence state is stable. In the general case, one has to consider the eigenvalues of the Jacobian matrix as in section .

Existence of stable coexistence region

Our assumptions of symmetry and normalization allow us to write

$$\omega_i(\boldsymbol{\rho}^a) = \frac{\psi(\rho_i^a)}{\sum_{j=1}^{n_l} \psi(\rho_j^a)} \tag{14}$$

where $\psi(\rho_i^a)$ is an arbitrary monotonically increasing function with $\psi(0) = 0$, which is bounded on the interval $[0, 1]$. We have

$$\left.\frac{\partial \omega_i}{\partial \rho_i}\right|_{\boldsymbol{\rho}^{a*}} = \frac{\psi'(\rho_i^{a*})}{\psi(\rho_i^{a*})} \frac{n_l - 1}{n_l^2} \tag{15}$$

which we can plug into Eq. (6) to obtain

$$\phi(\rho_i^{a*}) \equiv (1 - \rho_i^{a*}) \frac{\psi'(\rho_i^{a*})}{\psi(\rho_i^{a*})} < 1\,. \tag{16}$$

Since $\psi(0) = 0$ and $\psi'(0) \neq \psi(0)$, the left-hand side of Eq. (16) diverges for $\rho_i^{a*} \to 0$. Because ψ is bounded, we have

$$\lim_{\rho_i^{a*} \to 1} \phi(\rho_i^{a*}) = 0\,. \tag{17}$$

Therefore, there is always a ρ_i^{a*} (and so a value of λ) for which the inequality (16) is fulfilled.

[1] Kleineberg, K.-K. & Boguñá, M. Evolution of the digital society reveals balance between viral and mass media influence. *Phys. Rev. X* **4**, 031046 (2014).

[2] Gillespie, D. T. A general method for numerically simulating the stochastic time evolution of coupled chemical reactions. *Journal of Computational Physics* **22**, 403 – 434 (1976).

[3] Gillespie, D. T. Exact stochastic simulation of coupled chemical reactions. *The Journal of Physical Chemistry* **81**, 2340–2361 (1977).

[4] Doob, J. L. Markoff chains–denumerable case. *Transactions of the American Mathematical Society* **58**, pp. 455–473 (1945).
